# Chromatin Remodeling and Transcriptional Control in Innate Immunity: Emergence of Akirin2 as a Novel Player

**DOI:** 10.3390/biom5031618

**Published:** 2015-07-22

**Authors:** Sarang Tartey, Osamu Takeuchi

**Affiliations:** 1 Laboratory of Infection and Prevention, Institute for Virus research, Kyoto University, 53 Shogoin, Kawara-Cho, Sakyo-Ku, Kyoto 606-8507, Japan; E-Mail: startey@virus.kyoto-u.ac.jp; 2 AMED-CREST, Japan Agency for Medical Research and Development-Core Research for Engineering, Science, and Technology, Kyoto 606-8501, Japan

**Keywords:** macrophage, innate immunity, inflammation, transcriptional regulation, chromatin remodeling, NF-κB

## Abstract

Transcriptional regulation of inflammatory gene expression has been at the forefront of studies of innate immunity and is coordinately regulated by transcription factors, including NF-κB, and chromatin modifiers. The growing evidence for involvement of chromatin in the regulation of gene expression in innate immune cells, has uncovered an evolutionarily conserved role of microbial sensing and chromatin remodeling. Toll-like receptors and RIG-I-like receptors trigger these signaling pathways leading to transcriptional expression of a set of genes involved in inflammation. Tightly regulated control of this gene expression is a paramount, and often foremost, goal of most biological endeavors. In this review, we will discuss the recent progress about the molecular mechanisms governing control of pro-inflammatory gene expression by an evolutionarily conserved novel nuclear protein Akirin2 in macrophages and its emergence as an essential link between NF-κB and chromatin remodelers for transcriptional regulation.

## 1. Introduction

**Figure 1:**
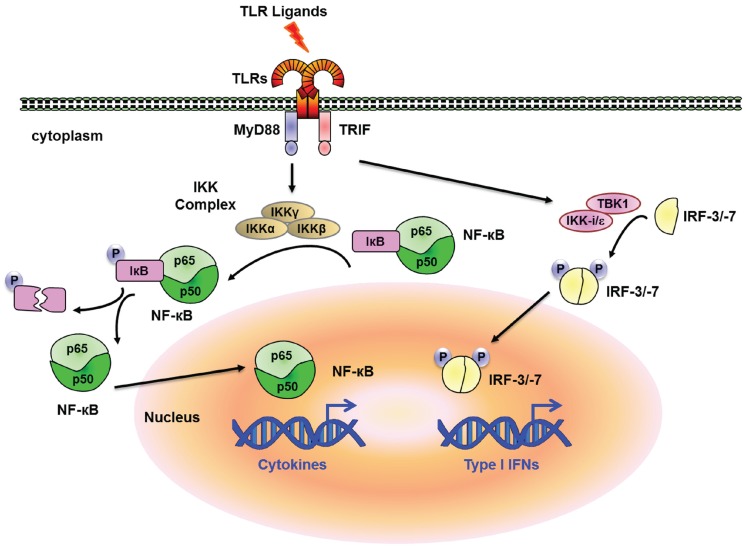
TLR Signaling and NF-κB activation. Stimulation of TLRs trigger the association of The Toll/interleukin-1 (IL-1)-receptor (TIR)-domain-containing adaptor molecule MyD88 which mediates the TLR-signaling pathway leading to the activation of the IKK complex, which consists of IKK-α, IKK-β and IKK-ɣ (also known as IKK1, IKK2 and nuclear factor-κB (NF-κB) essential modulator, NEMO, respectively). This pathway is used by TLR1, TLR2, TLR4, TLR5, TLR6, TLR7 and TLR9 and degradation of the phosphorylated IκB by the ubiquitin proteasome system allows the translocation of NF-κB to the nucleus where it activates the expression of cytokine genes (*Il6*, *Tnf*, *etc.*). Another TIR-domain-containing adaptor, TRIF, is essential for the MyD88-independent pathway. The non-typical IKKs IKK-ε and TBK1 mediate phosphorylation of IRF3/7 downstream of TRIF. This phosphorylated IRF3/7 translocates into the nucleus to induce expression of type I IFN and IFN inducible genes (*Ifnb*, *Cxcl10*, *etc*.).

Innate immune system senses molecular patterns from microorganisms and damaged cells [1,2]. These molecular patterns are recognized by several classes of sensor proteins, such as Toll-like receptors (TLRs), RIG-I-like receptors (RLRs), Nod-like receptors (NLRs) and so on. This innate immune system is highly conserved among different species from insects to mammals and has been extensively reviewed [3,4,5,6]. Once activated, these receptors trigger signalling cascades leading to transcriptional expression of a set of genes involved in inflammation [7]. Mobilization of these factors leads to tightly regulated changes in the expression of numerous genes involved in antimicrobial defence, phagocytosis, cell migration, tissue repair and the regulation of adaptive immunity [7,8,9,10,11,12]. Systemic inflammation is mediated by the action of proinflammatory cytokines and chemokines such as tumor necrosis factor (TNF), interleukin (IL)-1β, IL-6, IL-12, and type I interferons (IFNs). It has been well documented that transcription factors such as nuclear factor-κB (NF-κB), activator protein 1 (AP-1) and IFN-regulatory factors (IRFs) are critical for the expression of these inflammatory genes [13,14]. For instance, TLRs initiate the signaling cascades via adaptor proteins, myeloid differentiation primary-response protein 88 (MyD88) and/or TIR-domain-containing adaptor protein inducing IFN-β (TRIF), and activate distinct transcription factors, NF-κB and IRF-3/-7 via IKK (inhibitor of nuclear factor-κB (IκB)- kinase) and TBK1 (TRAF-family-member-associated NF-κB activator (TANK)-binding kinase 1)/IKK-*i*/ε, respectively (Figure 1). A dynamic and co-ordinately regulated gene expression programme lies at the heart of the inflammatory process. Whereas NF-κB is critical for the regulation of TLR-inducible genes in general, IRF-3/-7 regulate IFN-inducible genes in response to TLR ligands. This response empowers the host with a first line of defence against infection and the capacity to repair and restore damaged tissues. However, unchecked, prolonged or inappropriately scaled inflammation can be detrimental to the host and lead to several immune related disorders. These inflammatory responses are also critical for the pathogenesis of autoimmune diseases [7,15].

In all multicellular organisms, genes are co-ordinately regulated to control the activation or repression to ensure cellular homeostasis. In addition to a constant need to modulate the levels of expression of a large number of genes, mammalian cells are also faced with a topological challenge of packaging vast genetic information into the nucleus. Chromatin provides this unique ability to mammalian cells to package the DNA into these higher order structures [16,17]. This packaging of chromosomal DNA condenses and organizes the genome, but might obstruct several regulatory DNA elements. However, this constraint also allows nucleosomes and other chromatin components to actively participate in the regulation of transcription, chromosome segregation, DNA replication, and DNA repair. To enable dynamic access to packaged DNA and to tailor nucleosome composition in chromosomal regions, cells have evolved a set of specialized chromatin remodeling complexes (remodelers) [16,18,19,20,21]. The last decade has witnessed an unprecedented explosion of knowledge in the areas of chromatin remodeling complexes and epigenetic control of gene regulation in innate immune cells [22,23,24,25].

Despite all these advances in the field of transcription and chromatin biology, relatively little is understood as to the mechanism whereby developmentally critical transcription factors coordinate with chromatin remodeling factors to optimize target gene loci for gene expression. Such a mechanism might involve direct transcription factor/chromatin remodeling factor interactions, or could likely be mediated via an unknown intermediary. In this review, we will discuss recent progress concerning the molecular mechanisms governing control of inflammatory gene expression by an evolutionarily conserved novel nuclear protein Akirin2, and how this nuclear protein has emerged as an essential link between NF-κB and chromatin remodelers for transcriptional regulation.

## 2. Transcriptional Regulation and Chromatin Remodeling

Transcriptional regulation has always been at the frontline of the field of innate immunity. Although the importance of chromatin remodeling pathways in tumor biology and epigenetics has been accepted widely [26,27], recent studies are emerging to clarify the role of these pathways in the regulation of gene transcription. Despite more than ten years of research since the initial discovery of TLRs in the mammals, the mechanisms by which the transcriptional activation of NF-κB dependent genes from a distant promoter and transcription start site is governed remain poorly understood.

### 2.1. Influences of Histone Modifications on Transcriptional Activation

Although signal transduction pathways and transcription factors are clearly central mediators of the selective and stimulus specific responses, the transcriptional activation of mammalian genes requires chromatin modification or remodeling which plays a pivotal role in development, differentiation, inflammation and transcription in various organisms. It has long been known that chromatin alterations accompany the transcriptional activation of at least a subset of inducible genes. Transcriptional activators typically recruit enzymes that modify the tails of histones through acetylation, methylation, and phosphorylation [28,29,30]. However, a growing body of evidence suggests that the distinctive chromatin structure of individual genes and their regulatory elements plays a critical and active role in the selectivity of the response.

Albeit histone modifications, such as HAT (histone acetyl transferase) and HDAC (histone deacetylase) have been proposed to be involved in transcriptional activation and repression, respectively, decades ago, the consensus has begun to emerge in recent years. First, with the exception of methylation, histone modifications can change the net charge of nucleosomes, resulting in a loosening of DNA-histone interactions. This idea is in fact supported by the observation that acetylated histones are easier to displace from DNA both *in vivo* [31,32] and *in vitro* [33,34].

A number of histone acetylases have been identified during the last decade. They are quite diverse in terms of their enzymatic activity and regulation. p300/CBP coactivators were the first major known histone acetylases discovered and initially implicated in transcription, and later shown to enzymatically modify histones [35,36,37]. Two other transcriptional coactivators have also been demonstrated to be histone acetylases, ACTR (activator of the thyroid and retinoic acid receptor) and SRC-1 (steroid receptor coactivator). Both of them are involved in transcriptional activation by a variety of ligand bound nuclear hormone receptors [38,39,40], and p300/CBP are also known to bind to both ACTR and SRC-1. Therefore multiple histone acetylases are brought to the promoter by hormone receptors to activate transcription in a ligand-dependent fashion and the reason why multiple histone acetylases are required for activation is not yet clear.

### 2.2. Specificity of Chromatin Responses on Transcriptional Activation

Recent studies have suggested the involvement of chromatin to actively contribute to the selectivity of the response to a stimulus via cell type specific or stimulus specific manner [41,42,43,44]. These responses can diverge dramatically, though the differences between stimuli and cell types are often highly specific. This specificity is required to elicit innate and adaptive responses with the potential to successfully control the infection, via effectual cell-cell communication and the activation of appropriate effector mechanisms. Much of the specificity of the response results from the ability of different receptors and sensing mechanisms to activate different signaling pathways and transcription factors targeted by these pathways [7,45]. Although many proteins involved in innate immunity are expressed constitutively, others are expressed in an inducible manner. Such variability would confer variable requirements for chromatin modifying complexes whose recruitment and activities could be regulated in a stimulus-specific manner.

Albeit different stimuli can activate NF-κB in macrophages, each stimulus activates only a subset of the NF-κB target genes that are competent for activation [46,47]. Some potential target genes may not be activated by a given stimulus because, at these genes, NF-κB must bind cooperatively with another inducible transcription factor that is not induced by the stimulus [48]. However, other genes may not be activated because they possess a chromatin barrier that must be removed before NF-κB can gain access to its specific binding sites [49]. In order to overcome this chromatin barrier, a stimulus is needed to activate a signaling pathway that induces the expression or activity of a specific transcription factor that facilitates and recruits a chromatin remodeler to the promoter or enhancers of a subset of NF-κB target genes. Alternatively, the stimulus may itself induce the expression or activity of a nucleosome remodeling complex or histone modifying complex. Especially, if different genes possess different chromatin barriers, and if different signaling events are needed to remove the barriers, chromatin structure would have the potential to participate actively in the stimulus specificity of a response.

This stimulus specific transcriptional response can be broadly categorized into the primary response and the secondary response, although exceptions do exist [42,50,51]. Primary response genes requires transcription factors whose activities are induced by post-translational mechanisms in response to the initial stimulus, such as the nuclear translocation of NF-κB following phosphorylation and degradation of the cytoplasmic IκB-family inhibitors [52]. Transcription factors induced by post-translational mechanisms also frequently contribute to the activation of secondary response genes, but these genes require the activity of at least one protein that is newly synthesized during the primary response. Primary and secondary response genes can also be classified depending on their sensitivity to protein synthesis inhibitors, such as cycloheximide [46,47]. Newly synthesized proteins contributing to the activation of secondary response genes can be transcription factors, cytokines that act in an autocrine manner, receptors, or signaling proteins. Although inducible gene transcription requires inducible transcription factors, these factors can act together with ubiquitous and cell type—specific transcription factors at the promoters and enhancers of inducible genes [46].

## 3. Akirin2 Mediated Transcriptional Control of NF-κB Dependent Genes

### 3.1. Identification of Akirin as a Protein Involved in NF-κB-Dependent Transcription

The availability of powerful genetic tools to study *Drosophila melanogaster*, and evolutionary conservation of “Toll” signaling molecules (essential mediators of innate immunity in mammals) between mammals and *Drosophila*, led us to investigate the new molecules involved in the NF-κB-dependent transcription. We have previously identified CG8580 in an RNAi-mediated knockdown study and found that the knockdown of this gene led to impaired immune deficiency (Imd) pathway signaling and enhanced sensitivity of flies to Gram-negative bacterial infection [53]. We renamed this gene, “Akirin” (from the Japanese “akiraka ni suru”, which means “making things clear”) and found that it is highly conserved among orthologs identified throughout metazoa including *Drosophila*, mice, teleosts, and humans. The function of the Akirin protein has remained enigmatic; despite its high degree of conservation, it remains almost completely devoid of known functional domains, catalytic activity, or demonstrable DNA binding activity. Akirin has been reported to be crucial for regulating gene expression in a wide range of contexts [53,54,55]. It has also been established that Akirin has a pro-myogenic role during muscle regeneration in mice [56]. Humans and mice contain 2 *Akirin* family members (*Akirin1* and *Akirin2*) [53]. Gene targeting studies showed that *Akirin2* is required for embryonic development and the mice lacking this gene die by embryonic day 9.5. Fibroblast cells derived from *Akirin2*-deficient mouse embryos showed selective defects in NF-κB-dependent gene expression following stimulation through pathways involving the Toll-like receptor, interleukin-1 receptor or TNF receptor. All of these pathways converge on the activation of NF-κB. Furthermore, neither *Akirin1*-deficient mice nor cells derived from these animals have any obvious unusual characteristics [53]. However, the function of Akirin1 could be hidden through functional redundancy in the presence of Akirin2, a point that requires further investigation.

Akirin2 possesses two conserved regions at their N- and C-termini, the former containing a nuclear localization signal. It contains two conserved helical regions, one located in the central region (residues 75–95) and one in the C-terminus (residues 143–193), separated from each other and from the N-terminus by regions predicted to be intrinsically disordered. Neither of the conserved helical regions have significant sequence similarity to proteins of known function, but conserved intrinsically disordered regions are often important for mediating protein-protein interactions [25]. As Akirin sequences show no obvious DNA- or RNA-binding motifs, they represent a potential link between NF-κB induced transcription and upstream signaling.

Moreover, Akirin2 is also found to be critical for regulating histone modifications on its target gene promoters, such as *Il6 and Il12b* promoter region in response to lipopolysaccharide (LPS) in mouse macrophages as well as in HeLa cells. The acetylation of H3K9 as well as trimethylation of H3K4 on the *Il6* and *Il12b*, but not *Cxcl1* and *Tnf* promoters was induced in response to LPS in an Akirin2-dependent manner. These data suggested the involvement of promoter specific or stimulus specific responses in the chromatin remodeling which require Akirin2 [25].

### 3.2. Identification of Akirin2 Binding Partners and their Role in Immune Regulation

One mechanism whereby Akirin might function as a general cofactor for gene expression would be through interactions with chromatin remodeling complexes. In an independent study, Bonnay *et al.* provided a comprehensive view of Akirin function in NF-κB transcriptional selectivity during the innate immune response in *Drosophila* [24]. In an attempt to identify Akirin partners they have performed yeast two hybrid screening and found that BAP60, a component of the Brahma (SWI/SNF) ATP-dependent chromatin-remodeling complex, binds to Akirin upon immune challenge. In *Drosophila*, the Brahma complex associate with *Osa* forming the Osa-containing-SWI/SNF-like Brahma (BAP) complex, whereas an association with both Polybromo and BAP170 defines the PBAP complex. Each complex targets a mutually exclusive subset of Brahma-dependent genes [57,58]. They further show that the BAP, but not the PBAP, complex is required *in vivo* for efficient antimicrobial peptide synthesis and for the survival of flies following Gram-negative bacterial infection. Upon immune challenge, Akirin is able to bind Relish, forming a link between this transcription factor and the BAP complex on the promoter of a subset of NF-κB target genes. Relish-dependent genes thus fall into two groups, either relying on Akirin and the BAP complex (encoding mostly anti-microbial peptides (AMPs)), or expressing most of the negative regulators of the IMD pathway and AMPs independently of Akirin.

Mammals have a family of BAP60 homologues, comprised of BAF60a, BAF60b and BAF60c, which are encoded by the *SMARCD1*, *SMARCD2* and *SMARCD3* genes, respectively [59]. Akirin2 can interact with all three BAF60 proteins. Knockdown of all of the BAF60 proteins led to impaired *Il6* gene expression in response to LPS in the macrophage cell line, though BAF60b and BAF60c, but not BAF60a, were important for *Il6* gene expression in response to IL-1β in HeLa cells [25]. Although the function of BAF60a has not been well understood, down-regulation of BAF60b and BAF60c have been reported to reduce the expression of muscle creatine kinase (MCK) [60] and this peculiarity has been ascribed to the partial, functional redundancy between BAF60b and BAF60c [61]. Besides muscle cell development, BAF60c was also reported to be involved in lipogenesis [62]. Insulin signaling leads to the phosphorylation of BAF60c by atypical protein kinase C ζ/λ inducing its nuclear translocation and regulation of lipogenic genes. On the other hand, BAF60b was reported to interact with an E3 ubiquitin ligase Unkempt, and ubiquitination of BAF60b results in its degradation in a proteasome-dependent fashion [63]. In this regard, it is also interesting to explore if the BAF60 proteins undergo modification in the course of inflammation via Akirin2. Further studies are required to address differential involvement of the BAF60 proteins in controlling inflammation *in vivo*.

The hypothesis that Akirin2 acts in concert with NF-κB family members by physically associating with them, led to an important observation that *Il6* and *Il12b*, but not *Tnf*, mRNA expression to TLR stimulation requires an IκB-like molecule called IκB-ζ [64,65]. Another report suggested the involvement of IκB-ζ in the regulation of histone modifications, though the underlying mechanism is not well understood [66]. Our group has previously shown that IκB-ζ functions in the production of IFN-γ in natural killer (NK) cells by inducing histone modifications in response to IL-12 and IL-18 stimulation [67], suggesting that IκB-ζ also controls chromatin remodeling in NK cells. It has also been shown that IκB-ζ controls Th17 differentiation in T cells by regulating IL-17 gene expression via its promoter activity [68]. In contrast, IκB-ζ expressed in epithelial cells of lacrimal glands is critical for the prevention of their apoptotic cell death, which leads to a pathology similar to Sjogren’s syndrome [69].

Emergence of IκB-ζ as an Akirin2 binding protein via the C-terminal region of Akirin2 proved to be the missing link in this puzzle. Recent study also reported that IκB-ζ was required for the recruitment of Akirin2 as well as Brg1 to the *Il6* and *Il12b* promoters in macrophages in response to LPS. Reciprocally, Akirin2 is also required for the recruitment of IκB-ζ to the same promoters in response to LPS. The interaction between endogenous IκB-ζ and Akirin2 was also observed in resting macrophages. In addition, IκB-ζ was found to form a complex with BAF60 proteins via Akirin2 [25]. These data suggest that IκB-ζ is recruited to the LPS-inducible gene promoters together with Akirin2 and BAF60 proteins, where they induce chromatin remodeling. IκB-ζ has been shown to interact with NF-κB p50 subunit [65,70], and NF-κB p50 translocates from the cytoplasm to the nucleus upon IL-1β/TLR stimulation, indicating that the complex of IκB-ζ-Akirin2-SWI/SNF interacts with the NF-κB p50 subunit via IκB-ζ upon IL-1β/TLR stimulation and the whole complex is then recruited to the *Il6* and *Il12b* promoters.

Collectively, the IκB-ζ-Akirin2-BAF60 axis is critical to bridge the NF-κB and SWI/SNF complexes in innate immune cell activation. It is intriguing to speculate that IκB-ζ functions via Akirin2-mediated chromatin remodeling in various immune cell types. Further studies with conditional Akirin2-deficient mice will be required to uncover the broad functional roles of Akirin2 in various cells types *in vivo*.

### 3.3. SWI/SNF and CpG Island Dependency of Akirin Mediated Transcriptional Induction

Mammalian SWI/SNF complexes contain either of two catalytic subunits, Brg1 or Brm, which are associated with several accessory proteins, known as BAFs (Brg1-associated factors) [59,71,72,73]. Loss-of-function studies have revealed highly variable requirements for SWI/SNF complexes at LPS-induced genes in mouse macrophages [74,75]. Akirin2 shows one such example highlighting the variable requirement for SWI/SNF nucleosome remodeling complexes for transcriptional induction and that the selection of cell type and stimulus specificity is very tightly regulated. Notably, a majority of primary response genes were induced in a SWI/SNF-independent manner, whereas other primary response genes and most secondary response genes required SWI/SNF complexes for their transcriptional induction in the absence of Akirin2 in LPS-induced mouse macrophages.

**Figure 2:**
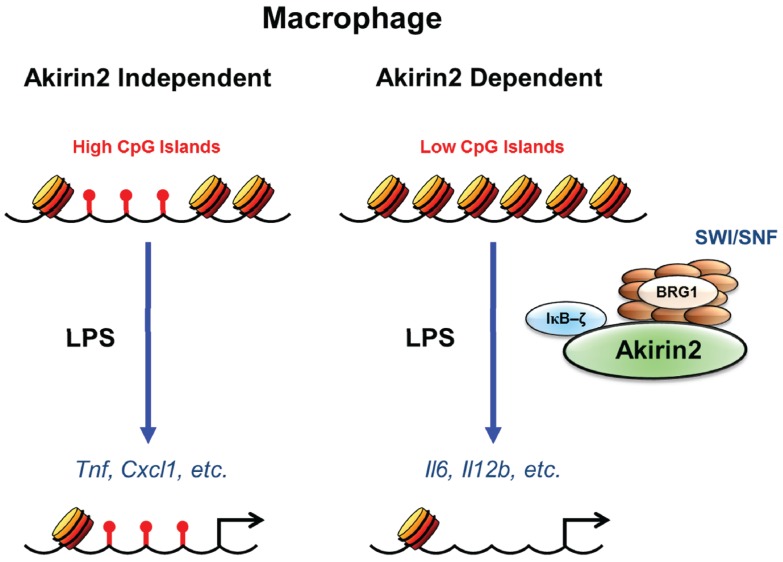
CpG islands and Akirin2 mediated transcriptional control. CpG island promoters are commonly found among genes which are transiently induced and also in the genes that are induced by a broad range of stimuli (such as *Tnf*, *Cxcl1*, *etc.*). These genes are proposed to have open chromatin and are Akirin2 independent which do not require chromatin remodeling for their transcription. In contrast, low CpG island promoters often require active nucleosome remodeling by SWI/SNF remodeling complexes following stimulation (such as *Il6*, *Il12b*, *etc.*). These low CpG island promoters have restricted chromatin access and consist of Akirin2 dependent genes requiring Akirin2 mediated recruitment of SWI/SNF remodeling complex for facilitating the gene transcription. These CpG island promoters can activate transcription factors, such as NF-κB upon activation by a broad range of stimuli and are frequently regulated by the stimulus specificity of a transcriptional response.

This SWI/SNF based selectivity was recently suggested to be dependent on the differential CpG island context of NF-κB target gene promoters, although there are contradictory studies. Most of the SWI/SNF-independent genes were characterized by CpG island promoters, whereas almost all SWI/SNF-dependent genes contained promoters with a low content of CpG dinucleotides [46,47]. Genome wide expression study in LPS stimulated Akirin2 deficient macrophages classified the Akirin2 dependent and independent genes based on the variable requirement of SWI/SNF complexes. However, the comparison of the promoter sequences of Akirin2-dependent and -independent genes did not reveal any significant differences in the frequency of NF-κB binding sites between Akirin2-dependent and -independent gene promoters. In contrast, the Akirin2-dependent genes tended to harbor significantly fewer CpG islands compared with Akirin2-independent genes. Recent advances in the field of epigenetics and chromatin regulation has revealed that the promoters containing fewer CpG islands are often assembled into nucleosomes that require active nucleosome remodeling by SWI/SNF complexes following stimulation. Furthermore, LPS-induced expression of *Cmpk2*, *Akap12* and *Parp14* was impaired in Brg1 knockdown cells, indicating that Akirin2 target genes require SWI/SNF complex for their expression irrespective of the presence of CpG islands on their promoter regions [25]. This remodeling requirement may restrict transcriptional activation to a more limited set of stimuli capable of activating factors that can recruit remodeling complexes to the specific gene promoters. In conclusion, among LPS-inducible genes, Akirin2 is essential to control expression of a set of genes whose promoter regions have relatively fewer CpG islands, suggesting emergence of a critical link between Akirin2 mediated transcription and chromatin remodeling (Figure 2).

An interesting feature of this evolutionary conservation stems from the observation that the genome of *Drosophila* is unmethylated and lacks classical CpG islands [76]. Even though *Drosophila* does not display CpG islands or methylation [77], bioinformatics analysis identified an enrichment of the CpG content in the sequences spanning the NF-κB target genes that are independent of Akirin and the SWI/SNF complex. In contrast, the promoters of Akirin and SWI/SNF-dependent genes are depleted of CpG-rich regions [24]. However, these data cannot be generalized as the authors have only analyzed immune genes. Nevertheless, it is tempting to speculate that, like CpG islands in vertebrates, CpG-rich sequences in *Drosophila* would establish regions of nucleosomal instability precluding any need of Akirin and the SWI/SNF complex for the control of gene transcription.

In addition to the control of innate immune responses, *Drosophila* Akirin has been shown to be required for Twist-mediated gene transcription by controlling chromatin remodeling during embryogenesis [55]. Akirin mutant embryos had muscle defects in the fly. Given that the lack of Akirin2 in mice results in early embryonic lethality, it will be interesting to investigate the role of Akirin2 in developmental tissues for controlling chromatin remodeling by coupling with various transcription factors. These results therefore strongly support the hypothesis that chromatin makes an active contribution to the stimulus selectivity of inducible transcription: The promoters of LPS-induced genes contain variable chromatin properties, which correlate with the variable requirement for SWI/SNF-dependent nucleosome remodeling. Moreover, this variability in chromatin properties and SWI/SNF dependence correlates, at least in part, with the extent to which genes must be regulated in a stimulus-selective manner.

Moreover, a recent study published using B cell specific Akirin2 mice, further emphasize the role of this enigmatic protein in various different cell types [78]. Having shown that chromatin remodeling in macrophages requires Akirin2 and its target gene expression is impaired in the absence of Akirin2 [25], authors additionally show that similar phenomenon can be observed in the B cells of Akirin2 mice and the B cells would also be defective in the recruitment of SWI/SNF complex upon its target gene promoters, thereby regulating B cell death and proliferation. Indeed, the ChIP analysis revealed that Brg1 is recruited to the putative-binding sites on the *Myc* and *Ccnd2* promoter (involved in various aspects of cell growth and proliferation) in response to CD40 stimulation in control B cells. In contrast, Akrin2 deficiency abrogated the recruitment of Brg1 to the *Myc* and *Ccnd2* promoter, indicating that Akirin2 is critical for the recruitment of the SWI/SNF complex to the *Myc* and *Ccnd2* promoter [78].

### 3.4. IFN-Inducible Gene Expression and Akirin2

In addition to the regulation of *Il6* and *Il12b* in response to stimulation with TLR ligands, Akirin2 was also found to be important for the induction of type I IFNs and a set of IFN-inducible genes in response to RNA virus infection [25]. Genome-wide microarray analysis suggested the presence of many IFN-inducible genes dependent on Akirin2 but not on IκB-ζ, suggesting that the induction of a set of IFN-inducible genes requires Akirin2-mediated chromatin remodeling, irrespective of IκB-ζ. Previous studies had shown that a nucleosome masks the TATA box and the transcription start site of the human IFNB promoter, and the action of SWI/SNF complex is required for this nucleosome sliding, enabling transcription [48]. Further, a set of IFN-inducible genes require chromatin remodeling by SWI/SNF, a complex that includes a specific subunit, BAF200 [79]. Since IFN-inducible genes have been suggested to be biased toward low CpG promoters and IFN-induced transcription factors promote nucleosome remodeling [80,81], it seems likely that Akirin2 is recruited to the IFN-inducible gene promoters independent of IκB-ζ for controlling chromatin remodeling. Since IκB-ζ is dispensable for the regulation of IFN-inducible genes such as Cxcl10 in macrophages in response to LPS [25], it is reasonable to speculate that Akirin2 controls the transcription of these genes by binding with yet unknown partners to recruit them to the specific promoter region. Further studies are required to elucidate all the mechanisms of IFN-inducible gene expression controlled by Akirin2.

## 4. Conclusions and Perspectives

Recent technological advances and the growing number of ChIP-Seq, and transcriptome profiling studies have enabled a great and rapid expansion of the knowledge of mechanisms controlling transcriptional responses to inflammatory stimuli. Availability of genome-scale expression data suggested that dynamic changes in gene transcription in innate immune cells are pivotal for evoking inflammation in response to pathogen infection. Gene expression is, in turn, controlled by elaborate and complex mechanisms.

**Figure 3:**
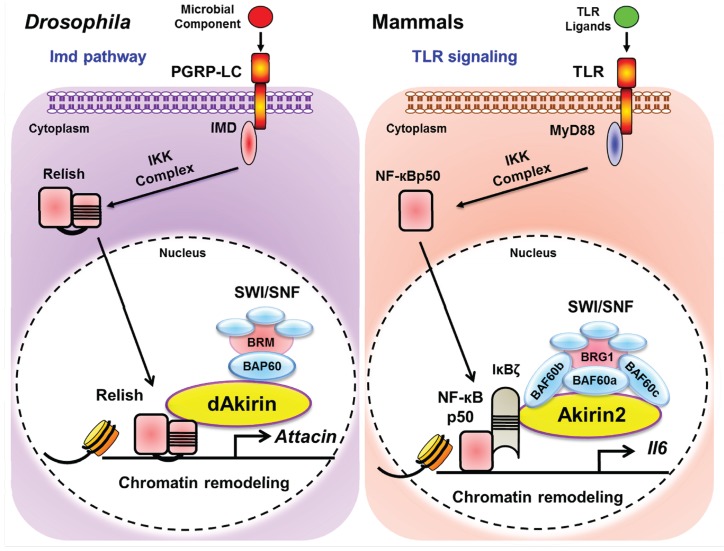
Evolutionary conservation of chromatin remodeling in inflammatory gene expression. (Left Panel) The *Drosophila melanogaster* Imd pathway. Peptidoglycan (PGN) from Gram-negative bacteria is sensed by PGRP-LC, which activate Imd. Imd recruits IKK signaling complex, which phosphorylates Relish, triggering its cleavage. The ankyrin repeats of Relish remain in the cytoplasm, and the “REL” moiety translocates to the cytoplasm to activate transcription of target genes. Akirin forms a complex with Bap60 (component of BRM complex) and facilitate the recruitment of whole complex to the promoters of its target genes. (Right Panel) The mammalian TLR signaling pathway. Activation of TLRs by their respective ligands leads to signaling that ultimately activates NF-κB. Similar to the process of the Imd pathway, IKK complex phosphorylates an inhibitory regulator of NF-κB, IκBα, resulting in release of NF-κB for translocation to the nucleus and activation of gene expression. p50 subunit of NF-κB interacts with the pre-formed complex of IκB-ζ-Akirin2-BAF60 (component of Brg1 complex) and facilitates the recruitment of SWI/SNF complex to the promoters of its target genes for their transcription.

Akirin2 represents a new class of transcription cofactor that bridges the transcriptional activation with those of chromatin remodeling complexes to influence gene expression. This mechanism is evolutionarily conserved and plays a pivotal role in regulating gene expression not only in mammals but also in insects (Figure 3). Targeting Akirin2 in immune cells might be beneficial for ameliorating inflammatory diseases and may lead to new strategies for combating autoimmune diseases. These results have opened new avenues of research that not only may help to unravel the complexities of the inflammatory signaling pathway in which Akirins function, but also may aid our understanding of the function of these molecules in embryonic development. The function of Akirins probably extend beyond the immune system, as do those of many other genes involved in immunity, and which also have roles in development. Future studies, aimed at the molecular mechanism of Akirin2 action in different cell types, as well as the identification of new Akirin2-dependent gene expression pathways will undoubtedly shed further light on this already fascinating yet enigmatic cofactor.
